# PP29 Contextualizing Gene Therapy Educational Resources For Hemophilia Patients In Brazil: Translation Process And Preliminary Qualitative Patient Preferences Findings

**DOI:** 10.1017/S0266462325102481

**Published:** 2025-12-29

**Authors:** Ana Dantas, Joana Balardin, Carla Fabrine Carvalho, Mariana Alves, Rafael Feitoza, Tania Maria Tania Maria Onzi Pietrobelli, Gabriela Yamaguti-Hayakawa, Daniela Pachito

## Abstract

**Introduction:**

Decision-making in the context of health technology assessment (HTA) of gene therapies for hemophilia needs to be tailored toward patients’ preferences. To promote equitable and patient-centered decision-making, it is crucial to provide patients with culturally attuned educational materials to ensure comprehension of key aspects of gene therapy so they can make legitimate and informed treatment decisions based on their preferences.

**Methods:**

In this mixed methods study, we used a contextualization approach to translate the educational tool from the PAVING study (Patient Preferences to Assess Value IN Gene Therapies in Hemophilia) into Brazilian Portuguese. Translated versions were reviewed by a hematologist, a patient association representative, and two industry professionals. The tool was subsequently piloted with two people with hemophilia (PWH). After revisions, the tool was virtually presented to 12 PWH to transfer knowledge in preparation for the interview about their willingness to receive gene therapy. They were also required to perform the attribute-ranking exercise from the PAVING study.

**Results:**

The translation process led to minor adaptations required for local context. The original version designed for computer use was adapted for use on a mobile phone. Pilot testing with PWH resulted in minor changes to optimize clickstreams. Ten participants indicated some baseline awareness of gene therapy for hemophilia (good n=2; reasonable n=3; bad or very bad n=5). Participants indicated a general willingness to receive gene therapy (very willing n=7; willing n=2; neutral n=3). The six top-ranked treatment attributes were “effect on factor level,” “dose frequency,” “impact on daily life,” “probability that prophylaxis can be stopped after treatment,” and “effect on annual bleeding rate.”

**Conclusions:**

These findings may contribute to further developments of the design and contextualization of hemophilia educational resources of gene therapy. The preliminary results on patient preferences will also inform future quantitative preference studies to be considered in the decision-making related to individual care and to reimbursement and coverage decisions in health systems, thus incorporating the patient perspective in HTA processes.

